# 

**Published:** 1886-03

**Authors:** 


					﻿SUBSCRIPTION PRICE, $3.00 PER YEAR.
COLLABORATORS.
CHICAGO:
Professor J. A. ALLEN, M. D., LL. D.
Professor E. ANDREWS, M. D., LL. D.
Professor W. H. BYFORD, A. M., M. D.
Professor O. C. DeWOLF, A. M., M. D.
Professor E. C. DUDLEY, A. B., M. D.
Professor J. H. ETHERIDGE, A. M., M. D.
Professor C. FENGER, M. D.
Professor J. N. HYDE, A. M.t M. D
Professor E. F. INGALS, A. M., M.D.
Professor H. A. JOHNSON, M. D., LL. D.
CHARLES GILMAN SMITH, A. M., M.D,
J. F. TODD, M. D.
MILWAUKEE, WISCONSIN:
Professor N. SENN, M. D.
WASHINGTON, D. C :
S. O. RICHEY, M. D.
UNITED STATES ARMY:
SURGEON, D. L. HUNTINGTON, M. D.
UNITED STATES NAVY:
MEDICAL DIRECTOR, J. M. BROWNE, M. D
MEDICAL DIRECTOR, A. L. GIHON, A. M.. M. D.
				

